# Experience of Peer Bloggers Using a Social Media Website for Adolescents With Depression or Anxiety: Proof-of-Concept Study

**DOI:** 10.2196/26183

**Published:** 2021-07-22

**Authors:** Sana Karim, Kimberly Hsiung, Maria Symonds, Ana Radovic

**Affiliations:** 1 Children's Hospital of Pittsburgh University of Pittsburgh Pittsburgh, PA United States; 2 University of Pittsburgh School of Medicine Pittsburgh, PA United States; 3 McLean-Franciscan Child and Adolescent Inpatient Program Franciscan Children's Brighton, MA United States; 4 Division of Adolescent and Young Adult Medicine University of Pittsburgh School of Medicine UPMC Children's Hospital of Pittsburgh Pittsburgh, PA United States

**Keywords:** adolescent, social media, blogging, depression, anxiety

## Abstract

**Background:**

Supporting Our Valued Adolescents (SOVA) is a moderated and anonymous social media website intervention. SOVA ambassadors are adolescents and young adults (AYA) asked to write monthly blog posts and comments on others’ posts on topics surrounding mental health.

**Objective:**

This study aims to understand the feasibility and acceptability of peer blogging for a moderated mental health intervention website and explore whether bloggers—AYA who self-report symptoms of depression and anxiety—experience potential benefits.

**Methods:**

AYA aged 14 to 26 years with a self-reported history of depression or anxiety were recruited to the SOVA Peer Ambassador Program. Participants were asked to write one blog post a month and comment at least four times a month on other blog posts, for which they were compensated for up to US $15 monthly. Outcome variables measured at baseline and 3 months after intervention included website usability and feasibility, depressive symptoms, anxiety symptoms, mental health treatment history, cybercoping, personal blogging style, self-esteem, loneliness, mental health stigma, social support, and positive youth development characteristics. Open-ended questions were asked about their blogging acceptability and usability.

**Results:**

Of 66 AYA showing interest and completing onboarding, 71% (47/66) wrote at least one blog post, with an average of 3 posts per person. A sample of 51% (34/66) of participants completed a 3-month survey for the full analysis. Almost all 34 participants were satisfied with the experience of blogging (32/34, 94%) and rated the website usability as good (80.1, SD 14.9). At 3 months, self-esteem scores increased by 2.1, with a small-medium effect size (*P*=.01; Cohen *d*=0.45), and youth competence and confidence increased by 0.7 (*P*=.002) and 1.3 (*P*=.002), with medium effect sizes (Cohen *d*=0.62 and 0.60), respectively.

**Conclusions:**

A blogging intervention for AYA with a history of depression or anxiety was feasible with regular and active engagement and shows evidence in a one-sample design for positive changes in strength-based assets—self-esteem, competence, and confidence—which map onto resilience.

## Introduction

### Background

Almost 12% of adolescents have depression and up to one-third have anxiety [1]. Suicidality contributes to US $12 billion in hospital costs [2], with one-third depressed adolescents experiencing suicidality and 11% having attempted suicide [3]. Less than half of the patients receive treatment [4], with initial treatment delayed by 10 years [5]. Less than one-fifth of adolescents with anxiety use mental health services [6].

Some of the most important barriers preventing adolescents from seeking help are a lack of mental health knowledge [7] and negative beliefs about treatment [8]. Despite these barriers, youth actively talk about experiences with depression and anxiety in web-based social environments [9-12], often seeking support [13,14]. A web-based environment may be best suited to reconsider negative health beliefs, as adolescents discuss their depressive symptoms on web [15,16], use social media for identity exploration [17], and for social norms setting [18]. This suggests that the web-based environment is a point of entry to begin talking about mental health symptoms, find support, and consider help-seeking.

**Figure 1:**
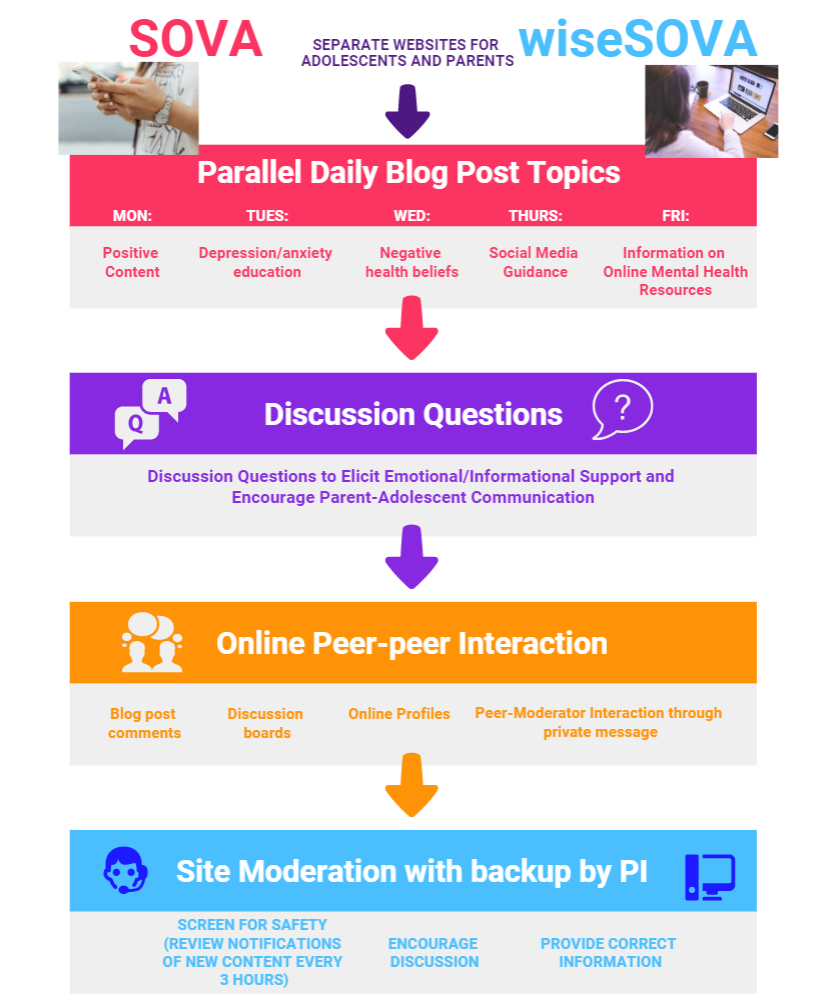
Supporting Our Valued Adolescents (SOVA) intervention design.

**Figure 2:**
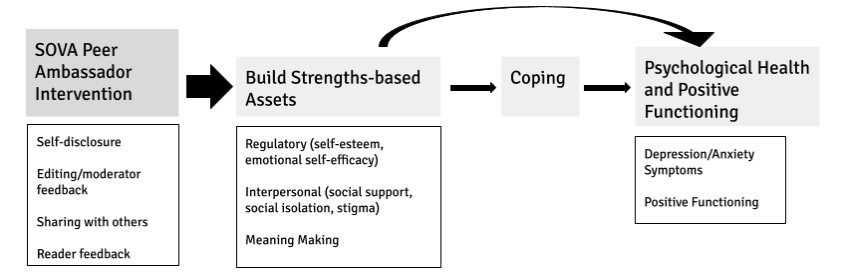
Supporting Our Valued Adolescents (SOVA) theory of change.

**Figure 3:**
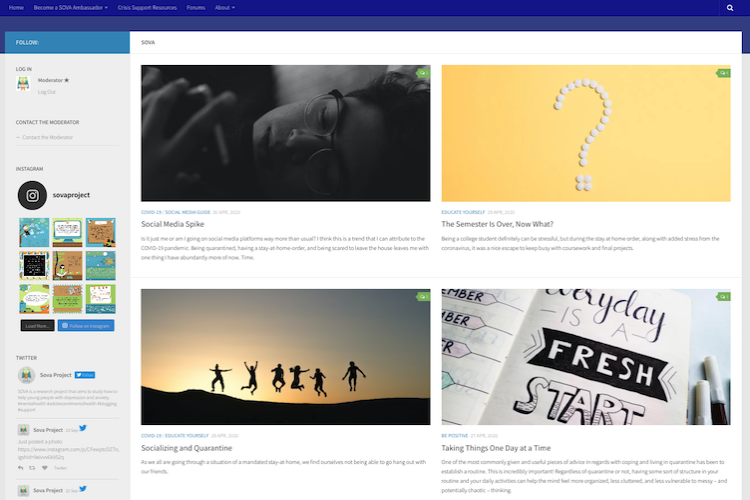
Preview of Supporting Our Valued Adolescents intervention (captured November 2020).

Supporting Our Valued Adolescents (SOVA) is a moderated web-based intervention designed to increase mental health knowledge, address negative health beliefs, and grow an anonymous web-based social support community for adolescents (Figure 1). The SOVA Blogging Ambassador Program is an accompanying intervention to SOVA, where participating adolescents and young adults (AYA) contribute authentic article content as a more interactive opportunity to offer peer support. Several observational studies found that individuals who write on web about their health experience improvement in social connectedness [19] related to self-disclosure [20] and an increase in meaning making [21], including making sense of illness [22]. Adolescents writing about peer difficulties as part of an experimental study had a decrease in their social-emotional difficulties when they wrote in a web blog open to reader commenting versus one that was closed [23]. The theory of change for the intervention can be viewed in Figure 2; a preview of the intervention can be viewed in Figure 3.

### Objectives

The SOVA Blogging Ambassador intervention offers opportunities for self-disclosure, sharing with others, and reader feedback. Reflecting on how to self-disclose a highly emotionally charged event in a readable way that is not self-defeating may help with regulatory strengths. Writing a post for readership or commenting on others’ posts may involve fostering web-based connections. Composing a post may involve making meaning of a lived experience to share with a reader audience and the capacity to find meaning, especially in difficult life events, can promote positive mental health [24]. This is especially relevant as many AYAs are experiencing multiple hardships because of the COVID-19 pandemic, including disruptions to their educational and career pursuits, increased rates of depression and anxiety, and social isolation [25].

This paper describes a single-arm exploratory trial of the SOVA Peer Ambassador Program to understand the feasibility and acceptability of monthly blogging for the SOVA sites, and to understand the initial benefits AYA with depression or anxiety may experience blogging. We specifically desired to understand the feasibility of engaging users to blog on the SOVA site, measured by blogging frequency; learn what prompted AYA to blog, and whether there were differences between those who showed interest but did not write blog posts and those who did write blog posts; and explore whether SOVA ambassadors experience psychological benefits or resilience, specifically examining depressive symptoms, anxiety symptoms, self-esteem, emotional self-efficacy, social support, social isolation, and stigma.

## Methods

### Recruitment

Approval for the study was obtained from the University of Pittsburgh Institutional Review Board. Participants were recruited using various methods, including fliers and posted advertisements at an academic medical center AYA clinic and local college campuses and the University of Pittsburgh’s web-based recruitment database Pitt+Me. Study advertisements were posted on the Instagram page (@sovaproject) and distributed during community mental health conferences and presentations.

Once participants emailed confirming an interest in the study, they created an anonymous username on SOVA and completed a screening survey to determine eligibility. Potential participants were included if they were between the ages of 14 and 26 (inclusive), capable of reading and writing English, completed the sixth grade, had internet and email access, and self-reported current or prior symptoms of anxiety or depression. Those with severe social isolation scores and depressive symptoms indicated on the Patient Health Questionnaire-9 (PHQ-9) were allowed to participate, but those who endorsed suicidality with the intention to act or had a history of a suicide attempt with no subsequent follow-up treatment were excluded from the study and provided crisis resources.

Those who qualified for the study completed a web-based consent form and confirmed that they were interested in continuing. Parental permission was waived by the Institutional Review Board to allow those between 14 and 17 to easily participate because of minimal risk and because some parents may be unaware of their child’s mental health symptoms. Participants were then sent a web-based baseline survey through Qualtrics.

After completing the baseline survey, the SOVA Peer Ambassador Advisor arranged a meeting time to explain the blogging process over the phone to complete onboarding. The 10-minute call provided an opportunity for participants to ask questions, reminded the participant that blogging was not a replacement for therapy, and covered the study protocol as detailed below.

Participants were encouraged to write one blog post a month on any mental health topic. These posts were classified into four categories: *Be Positive, Educate Yourself, Social Media Guide, and Links.* When participants began writing for the website, there were already about 160 articles published by the research team to help set a standard for writing style and web-based community norms. All blog posts were reviewed for factual accuracy, sensitive content, and grammar. Feedback was provided via email from the advisor. The advisor and site moderators of the SOVA website were all trained to address blog posts or comments posted by participants, which suggested that they were at risk for harming themselves or others.

Participants were encouraged to leave comments on other blog posts on SOVA. These could be in response to the questions at the end of the blog post by the moderator to elicit comments, or about whatever they wished to respond to another participant’s blog post. Participant blog posts were uploaded to the SOVA website on weekdays, excluding major holidays, along with posts written by the research team. Participants were sent a postsurvey via email, 3 months after the phone call. Participants received compensation on a prepaid debit card based on how frequently they contributed to the website. They received US $10 if they wrote a blog post a month and an additional US $5 if 4 comments were left on the website in the same month. Participants were paid US $10 upon completion of the 3-month postsurvey. There was no official duration; participants could withdraw at any time and when they turned 27 and aged out of the study.

### Measures

#### Demographics

The baseline survey included questions about age, gender identity, sexual orientation, socioeconomic status [26], race, and ethnicity. Demographics were not initially recorded to ensure full anonymity for the study but were added in July 2019. Because this sample included participants who joined before July 2019, they will not be included in the analysis for this study.

#### Usability

The System Usability Scale [27] (Cronbach α=.88) is a robust and versatile tool used to assess users’ subjective ratings of product usability. Ten items are rated on a 5-point Likert scale (1=strongly disagree to 5=strongly agree), and scores were scaled to a range of 0-100 [28]. The scale demonstrated strong structural validity and reliability using over 2000 surveys compiled from 206 studies. The mean usability score of the pool was 70.

Additional single-item questions were asked to assess feasibility. User-friendliness was assessed through the question, “Overall you would rate the user-friendliness of this site as:” followed by 7 response options (1=worst imaginable; 7=best imaginable). Dichotomous items include, “Were you satisfied with the experience of blogging for this project?,” “Did you access the website?,” and “Did you gain something from blogging?” All feasibility questions were asked at 3 months.

#### Depressive Symptoms

The PHQ-9 (Cronbach α=.86), modified for adolescents, is a 9-item diagnostic tool used to assess depression severity [29,30]. Each item of the questionnaire is scored on a 4-point Likert scale (0=not at all; 3=nearly every day) with different scores indicating different levels of depression (5-9: mild, 10-14: moderate, 15-19: moderately severe, and ≥20: severe). A score of ≥11 was considered a positive screen for clinically significant depression. The scale included 2 additional questions: “In the past year have you felt depressed or sad most days, even if you felt okay sometimes?” that had dichotomous response choices and “If you checked off any problems, how difficult have these problems made it for you to do your work, take care of things at home, or get along with other people?” The second question was scored on a 4-point Likert scale (1=not difficult at all; 4=extremely difficult). The PHQ-9 has been found to be a reliable and valid measure of depression severity [29], and the PHQ-9 has been validated for diagnostic accuracy in this age group [30].

#### Anxiety Symptoms

Anxiety symptoms were assessed using the Screen for Child Anxiety Related Emotional Disorders—Child Version—5 (Cronbach α=.52), a 5-item short-form self-report screening tool for childhood anxiety disorders [31]. Items were rated on a 0-2 point rating scale, with 0=not true or hardly ever true, 1=somewhat true or sometimes true, and 2=very true or often true. Total scores of ≥3 were considered as a positive screen for clinically significant anxiety with 74% sensitivity and 73% specificity. The Screen for Child Anxiety Related Emotional Disorders—Child Version—5 has demonstrated adequate validity for use in screening anxiety disorders in community settings [31].

#### Personal Blogging Style

The Personal Blogging Style Scale [32] is a 25-item scale used to characterize individuals’ blogging styles as therapeutic (Cronbach α=.58; directed to concerns of bloggers than blog readers), substitution (Cronbach α=.62; focus on interaction with others as a substitute for social networks), self-censoring (Cronbach α=.13; focus on positive portrayal and self-presentation over open communication), and connected (Cronbach α=.29; focusing on connecting and communicating with others rather than solving emotional problems). Only participants who reported having written blogs before completed this scale. Response options used a 5-point Likert scale (1=completely disagree; 5=completely agree). Scores were calculated by summing the relevant item ratings. The Personal Blogging Style Scale has been found to have good validity and reliability in identifying blogging styles [32].

#### Cybercoping

Cybercoping, or the act of problem solving in cyberspace, was assessed using items from the Developing Coping Skills Online scale, a 30-item scale used to assess web-based coping skills of those with chronic diseases [33]. As mental health conditions are highly comorbid with chronic physical illnesses [34,35], carry a heavy global burden of disease [36], and are considered to be a chronic condition by the Centers for Medicare and Medicaid Services [37], this scale was determined to be suitable for the study. Only patients who reported having read blogs before completed this scale. Domains deemed relevant by the study authors include enhancement of emotion-focused coping (6 items; Cronbach α=.92), enhancement of problem-focused coping (5 items; Cronbach α=.71), and affective coping outcome (4 items; Cronbach α=.93); domains that were not included were those that pertained to physical diseases. Response options used a 7-point scale (1=not at all; 7=very much). Scores were summed, with higher scores indicating higher coping capabilities and outcomes. The original 30-item scale demonstrated adequate structural and construct validity among individuals with chronic conditions, including depression [33].

#### Self-esteem

The 10-item Rosenberg Self-Esteem Scale (RSES) [38] (Cronbach α=.89) was administered to assess participants’ self-esteem. Items on the RSES ask about self-worth and self-acceptance and are scored using a 4-point scale (1=strongly disagree; 4=strongly agree). The scores on each question were summed together, with higher scores indicating greater self-esteem. The RSES is the most widely used measure of global self-esteem in the literature [39] and demonstrates concurrent, predictive, and construct validity with significant correlations with other measures of self-esteem and predictive measures of depression and anxiety [40].

#### Emotional Self-efficacy

Emotional self-efficacy was assessed using the Mental Health Self-Efficacy Scale (MHSES; Cronbach α=.74). The MHSES was developed according to Bandura guidelines to create self-efficacy questionnaires [41]. The questionnaire contains 5 items asking about the participants’ confidence level in performing mental health self-care behaviors. Scoring of the MHSES is based on a 5-point Likert scale (0=disagree very much; 5=agree very much).

#### Social Isolation

The revised University of California, Los Angeles Loneliness Scale was administered to all participants to measure social isolation (Cronbach α=.93). This 20-item scale measures one’s feelings of social isolation and is scored using a 4-point scale (1=never; 4=often) [42]. The revised measure was updated to counter the possible effects of response bias and was shown to have evidence of concurrent and discriminant validity among college students [42].

#### Perceived Stigma

Perceived stigma was assessed using the 9-item Depression Stigma Scale and perceived stigma factor [43] (Cronbach α=.93). Items of the scale touch on different themes about depression, such as the extent to which depression is an illness, how much control people have over their depression, and the degree to which depression is seen as a character flaw or something that should not be discussed. Using a 5-point Likert scale (0=strongly disagree; 4=strongly agree), perceived stigma was measured by asking participants to rate their agreement with the statements based on what they think other people believe. The higher the score on the scale, the greater the stigma that a person has. The measure has been shown to have adequate internal consistency (Cronbach α=.82) and test-retest reliability in a population of individuals with depressive symptoms [43].

#### Social Support

The eight-item emotional and informational support subscale from the Medical Outcomes Study Social Support Survey [44] (Cronbach α=.93) was administered to participants to assess social support. This scale measures the level of emotional and informational support available to the participants. Each item is rated using a 5-point scale (1=none of the time; 5=all of the time). The Medical Outcomes Study Social Support Survey demonstrates reliability among a population of chronically ill patients and has been found to be fairly stable over time [44].

#### Positive Youth Development

Positive youth development was assessed using the Positive Youth Development Very Short Form (PYD-VSF) [45]. The PYD-VSF is a questionnaire that measures adolescent strengths based on the Lerner and Lerner Five Cs of PYD [46]: competence (3 items, total score ranging from 3-12), connection (4 items, total score ranging from 4 to 20), confidence (3 items, total score ranging from 3 to 13), caring (3 items, total score ranging from 3 to 15), and character (4 items, total score ranging from 4 to 19). Items were heterogeneous in their response formats (ie, a mix of 4-point and 5-point scales). The PYD-VSF was shown to have structural validity evidence that ran parallel with its derivative, the PYD-Short Form, among a population of adolescents [45].

#### Participant History

Two additional questions were asked at both baseline and after 3 months regarding treatment history (“Have you ever received help from a professional psychologist or counselor for any personal or emotional problems you have experienced?” and “Have you ever used medication like an antidepressant for any personal or emotional problems you have experienced?”) with dichotomous response options. At baseline, two additional questions were asked regarding blogging history (“Have you read a blog to help you understand your mental illness?” and “Have you ever written a blog? (y or n) Please describe below”).

#### Open-ended Responses

Participants were invited to answer three open-ended questions at the baseline. All participants were asked, “Why are you interested in blogging.” The other two open-ended questions asked participants about their experience in reading blogs to understand mental illness and write blogs if they answered “yes” to these questions about blogging history, as mentioned in the previous section.

In the 3-month survey, participants were invited to respond to eight open-ended questions about their experience with the website and if they had any feedback. Questions included the following: what prompted you to log on, why were you satisfied with the website, why were you not satisfied with the website, what were your reasons for continuing with the study, what worries did you have, what did you gain from the website, what did you like about the website, and what would you change about the website?

Having a space for participants to provide open-ended responses in their own words gave researchers a more detailed opportunity to review participant background and interest in both blogging and SOVA, as well as a space to give candid feedback.

### Analysis

Baseline descriptive statistics were reported using means (SD) for continuous outcomes and frequencies (%) for dichotomous outcomes. The following comparisons were made: preintervention (baseline) and postintervention (3 months), baseline comparisons between bloggers who completed the 3-month survey and those who did not, baseline comparisons between those who blogged and those who did not, and baseline data between those who completed the 3-month survey and those who did not. These were performed using paired two-tailed *t* tests for continuous variables and McNemar test for dichotomous variables. Effect sizes were calculated using: Cohen *d*=mean of the difference/SD of difference [47]. To detect the actual blogging impact, a sensitivity analysis was performed by removing participants who did not blog (ie, nonbloggers) and then rerunning the paired comparisons. Model diagnostics showed no need for transformation of any variables. Missing data were not included in the *t* test. *P* values represent two-sided tests; results were statistically significant at *P*<.05.

Text-based responses to open-ended questions were manually and individually reviewed and coded once to find any common themes by the first and third authors using content analysis and the approach of qualitative description as proposed by Sandelowski [48]. After reviewing both sets of codes, the first author developed a codebook for both baseline and 3-month free-text responses. These codes were then reviewed and discussed with the remaining authors to increase their validity. None of the authors reported any discrepancies, and there were no disagreements.

## Results

### Blogger Information and Participation

**Figure 4:**
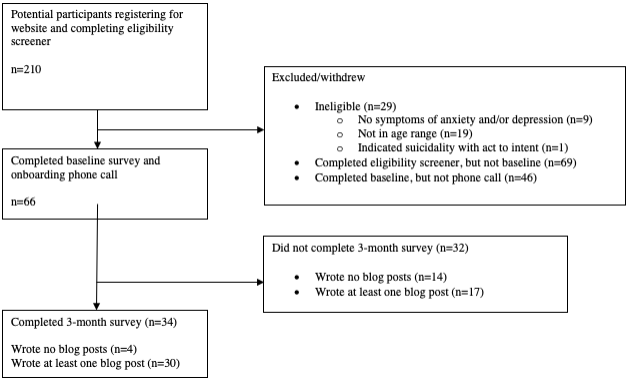
Strengthening the reporting of observational studies in epidemiology recruitment diagram.

Overall, 66 AYA completed the onboarding process between April 2018 and July 2020, which included completing the baseline survey (full recruitment numbers are available in Figure 4). In this sample, 71% (47/66) of participants completed at least one blog post. In addition, 52% (34/66) completed the full 3-month survey. Participants were categorized into bloggers (ie, participants who wrote at least one blog post during the study) and nonbloggers (ie, those who did not contribute to any blog posts). There were no significant differences at baseline between bloggers and nonbloggers (Table 1), between completers and noncompleters of the 3-month survey, or between bloggers who completed the 3-month survey and those bloggers who did not, although data missingness greatly limits our interpretation of any differences.

Participants who blogged had an average age of 20 years (SD 3.2); 40% (19/47) had a PHQ-9 score consistent with depression, whereas 87% (41/47) had a SCARED-C score consistent with anxiety. Most bloggers (40/47, 85%) had previously been seen by a professional psychologist or counselor, and 60% (28/47) had taken medications such as antidepressants.

No concerning blog posts or comments during the studied time period required reaching out to emergency contacts and sending crisis resources. There was only one blog post that was not posted; this was because of applying misinterpretations of bipolar disorder onto themselves. The participant was contacted and a moderator explained that the post had factually incorrect information, directing them to more information about bipolar disorder. The participants wrote a new post for that month instead.

**Table 1:** Comparison of baseline data between bloggers and nonbloggers.

Outcome			Bloggers (n=47)					Nonbloggers (n=18)					*P* value
			Value, mean (SE)		Participant, n (%)^a^		Value, mean (SEM)			Participant, n (%)^a^			
**Depressive symptoms**													
	PHQ-9^b^ score (range 0-27)	9.8 (0.8)		N/A^c^		12.8 (1.4)			N/A		.06		
	PHQ-9 score consistent with depression (range≥11)	N/A		19 (40)		N/A			9 (50)		.53		
**Anxious symptoms**													
	SCARED-C^d^ score (range 0-15)	4.4 (0.3)		N/A		4.8 (0.7)			N/A		.62		
	SCARED score consistent with anxiety (range≥3)	N/A		41 (87)		N/A			13 (72)		.09		
**Treatment history (having ever received)**													
	Ever seen professional psychologist or counselor	N/A		40 (85)		N/A			17 (94)		.39		
	Ever taken medication like antidepressants	N/A		28 (60)		N/A			12 (67)		.67		
	Cybercoping (range 15-105)^e^	58.2 (4.4)		N/A		68.1 (6.6)			N/A		.22		
	Self-esteem (range 10-40)	25.6 (0.8)		N/A		26.1 (1.4)			N/A		.73		
	Mental health self-efficacy (range 0-20)	16.0 (0.4)		N/A		16.5 (0.8)			N/A		.56		
	Social isolation (range 20-80)	43.2 (1.6)		N/A		46.2 (2.5)			N/A		.32		
	Perceived stigma (range 0-36)	22.7 (1.2)		N/A		21.2 (1.9)			N/A		.54		
	Social support (range 8-40)	31.0 (0.9)		N/A		28.7 (1.7)			N/A		.19		
**Positive youth development**													
	Competence (range 3-12)	7.6 (0.2)		N/A		7.6 (0.4)			N/A		.88		
	Confidence (range 3-13)	7.0 (0.4)		N/A		7.7 (0.6)			N/A		.34		
	Character (range 3-19)	14.7 (0.6)		N/A		15.7 (0.8)			N/A		.47		
	Caring (range 3-15)	14.0 (0.2)		N/A		14.2 (0.3)			N/A		.68		
	Connection (range 4-20)	14.0 (0.5)		N/A		13.1 (0.6)			N/A		.28		

^a^Calculations used exact sample sizes so that missing data were not included.

^b^PHQ-9: Patient Health Questionnaire-9.

^c^N/A: not applicable.

^d^SCARED-C: Screen for Child Anxiety Related Disorders-Child.

^e^Sample size is out of those who reported having read blogs before (ie, n=19 for bloggers, n=9 for nonbloggers).

### Feasibility

Between April 2018 and July 2020, 31.8% (188/591) of the published blog posts were written by participants. There were no examples of technical difficulties from the participants regarding website use.

In the 3-month survey, 68% (23/34) reported logging on less than once per week, and 32% (11/34) logged on at least weekly. We analyzed and coded 272 free-response responses in the 3-month survey. The codebook for free-response questions in the 3-month survey is presented in Table 2. Participants were primarily prompted to log onto the website to engage with the website by reading the blogs, writing and responding to comments, and writing their blog. Others were prompted to fulfill the study requirements. No one was prompted because of external triggers, except for one participant who had set up calendar reminders to go on the website. Reasons provided for why they continued to blog included out of enjoyment for writing and being a part of and helping the website community. Other frequent reasons included having a space to vent and write to cope and for the monetary compensation.

**Table 2:** Three-month free-response codebook.

Category and code		Definition	Example
**Community**			
	Not feeling alone	Participant mentions that the intervention has them relating to blog posts and feeling that what they are going through is not just happening to them	“I liked that I was able to connect with people similar to me.” [ID 41]
	Rejection from others	Participant worries that other participants will not enjoy, comment, or will judge their posts	“I worried whether or not people would appreciate what I had to say or if they would just brush it off as if I didn’t know what I was talking about.” [ID 63]
	Helping others	Participant mentions using, continuing, or enjoy the intervention because they use their experience and posts to support others	“I want to help other people like me who suffer from mental illnesses” [ID 51]
	Community support	Participant explicitly mentioned feeling a sense of community with other participants using intervention	“I gained a sense of community and support from people from all backgrounds. It is nice to be able to be vulnerable without the fear of being invalidated.” [ID 44]
Writing		Participants indicated an interest in writing and writing skills, whether it was writing in general or the process of writing for the intervention	“I was able to improve my writing by blogging.” [ID 69]
**Barriers to blogging**			
	Time	Participant did not have enough time to use the intervention	“My primary worry is time, I am an incredibly busy college student and I was not sure I would have time to make comments and write blogs.” [ID 106]
	Ideas	Participant expressed worries about not having something to write about	“Maybe that I would run out of ideas.” [ID 64]
**Benefits to self**			
	Self-reflection	Participant enjoyed using the platform because it gave them an opportunity to reflect on their experiences	“Insight to my own strengths and weaknesses, tips from others, a sense of community.” [ID 67]
	Education and resources	Participant learned something by reading other blog posts or mentioned access to resources listed on website	“I’ve learned a LOT of information from reading other people’s posts, including multiple apps/websites that I now use, which is just really cool and something that I didn’t initially expect to get out of this experience.” [ID 47]
	Outlet	Participant mentioned that the intervention served as a place for them to openly talk about what they were going through	“It may be because of covid, but my mental health has been a rollercoaster, so I needed this blog to feel sane. The blogs have really helped me have a space to let out how I feel, and also learn from others.” [ID 96]
**Anonymity**			
	Benefit	Participant stated that they enjoyed the anonymity or confidentiality of the intervention	“I could be anonymous if I wanted to and I could speak my mind and see others who felt the same way.” [ID 66]
	Fear of losing anonymity	Participant expressed worry that someone could trace their blogs back to them	“I was worried that my blogs would be traced back to me. I would prefer to stay anonymous.” [ID 73]
Mental health effect		Participant mentions any impact that the intervention had on their mental health	“I’ve gained better a perspective about mental health and the work that I am doing with my therapist.” [ID 103]
Critiques		Criticisms, feedback, and recommendations that participants had about the intervention	“It took me a while to figure out how to blog as well as navigate the site.” [ID 90]

The primary concerns that participants had about continuing to blog were time constraints and busyness that at times prevented them from contributing and feeling pressured to blog in addition to these constraints. Some were also concerned with running out of ideas and whether other users would like their content. A few participants mentioned worries about the website itself, with several mentioning that they were afraid of the risk of losing their anonymity.

Almost all (32/34, 94%) of the participants at 3 months were satisfied with their blogging experience. The reasons included the simplicity of the website, having a place to express themselves, receiving support from moderators and other users, and helping others. One user stated as follows:

If I were in high school again and I was able to see where I’d be five or ten years from that age, I would be amazed at my progress and would feel so hopeful about my future. This blog is successful in making that connection, and I fully support it’s mission.

ID 63

A total of 82% (28/34) of participants reported having gained something from blogging. The overall mean usability score was 80.1(SD 14.9), 13% higher than that of the initial usability study [49]. The overall mean user-friendliness rating was 5.3(SD 0.8), consistent with good or excellent. When asked about what they gained, the majority of participants felt more comfortable, confident, and reassured about their writing abilities and feelings. Participants felt that they had gained considerable new information from the website.

Participants provided feedback on the design and function of the website. Many were satisfied with the variety of articles and the website’s organization, navigability, and accessibility. Although 21% (7/34) said that they would not change anything about the website, the most common critique was that the website was difficult to navigate at first and wanted the site to be more interactive. Suggestions for site changes included a discussion board, a suggestion box for blog topics, and weekly polls.

### Pre- and Postintervention Comparison

Baseline and 3-month outcome comparisons are presented in Table 3. For the full sample, self-esteem scores increased at 3 months by 2.2, with a small-medium effect size (*P*=.01; Cohen *d*=0.45), and youth competence and confidence increased by 0.7 (*P*=.002) and 1.3 (*P*=.002), with medium effect sizes (Cohen *d*=0.62 and 0.6), respectively.

**Table 3:** Baseline and 3-month outcome comparison.

Outcome		Baseline (N=34)		3 months (N=34)		Difference^a^		*P* value
		Value, mean (SE)	Participant, n (%)^b^	Value, mean (SE)	Participant, n (%)^b^	Value, mean (SE)	Participants, n	
**Depressive symptoms**								
	PHQ-9^c^ score (range 0-27)	9.8 (1.1)	N/A^d^	9.0 (0.7)	N/A	0.8 (0.9)	N/A	.41
	PHQ-9 score consistent with depression (range≥11)	N/A	11 (32)	N/A	10 (29)	N/A	–1	.99
**Anxious symptoms**								
	SCARED-C^e^ score (range 0-15)	4.5 (0.4)	N/A	4.1 (0.4)	N/A	–0.4 (0.3)	N/A	.22
	SCARED score consistent with anxiety (range≥3)	N/A	28 (82)	N/A	25 (74)	N/A	–3	.45
**Treatment history (having ever received)**								
	Ever seen professional psychologist or counselor	N/A	29 (86)	N/A	28 (82)	N/A	–1	.99
	Ever taken medication like antidepressant	N/A	17 (50)	N/A	19 (56)	N/A	2	.50
	Cybercoping (range 15-105)	63.3 (6.6)	N/A	59.7 (6.4)	N/A	–3.6 (5.1)	N/A	.50
	Self-esteem (range 10-40)	25.9 (1.0)	N/A	28.0 (0.9)	N/A	2.1 (0.8)	N/A	.01
	Mental health self-efficacy (range 0-20)	15.8 (0.5)	N/A	16.1 (0.5)	N/A	0.3 (0.5)	N/A	.58
	Social isolation (range 20-80)	43.5 (2.1)	N/A	40.0 (2.1)	N/A	–3.5 (1.8)	N/A	.06
	Perceived stigma (range 0-36)	23.3 (1.6)	N/A	21.1 (1.5)	N/A	–2.2 (1.2)	N/A	.08
	Social support (range 8-40)	30.9 (1.1)	N/A	31.9 (1.1)	N/A	1.0 (1.0)	N/A	.35
**Positive youth development**								
	Competence (range 3-12)	7.6 (0.3)	N/A	8.3 (0.3)	N/A	0.7 (0.2)	N/A	.002
	Confidence (range 3-13)	7.0 (0.5)	N/A	8.3 (0.4)	N/A	1.3 (0.4)	N/A	.002
	Character (range 3-19)	14.4 (0.8)	N/A	15.6 (0.4)	N/A	–0.5 (0.4)	N/A	.19
	Caring (range 3-15)	14.0 (0.2)	N/A	14.2 (0.2)	N/A	0.2 (0.3)	N/A	.52
	Connection (range 4-20)	14.2 (0.6)	N/A	13.8 (0.5)	N/A	–0.5 (0.5)	N/A	.40

^a^Difference is expressed as the difference in mean (SE of the mean) for continuous variables and the difference in quantity for dichotomous variables.

^b^Calculations used exact sample sizes so that missing data were not included.

^c^PHQ-9: Patient Health Questionnaire-9.

^d^N/A: not applicable.

^e^SCARED-C: Screen for Child Anxiety Related Disorders-Child.

## Discussion

### Principal Findings

The SOVA intervention was designed to provide adolescents with mental illness symptoms an opportunity to blog and comment about mental health in an anonymous, peer-supported, and moderated space. The results of the surveys taken at baseline and at 3 months answered some of the initial research questions designed before the study, including the website’s feasibility and usability, reasons to initially blog and continue to blog, and the effects on bloggers’ mental health. First, the website had favorable feasibility and usability ratings. Second, participants primarily answered that they enjoyed writing and wanting to help others when asked why they continued to blog and participate in the intervention. This did not change after 3 months. In fact, after using the website, participants reported that they were satisfied with the blog and continued to do so because of the sense of community and they were able to help others and share their own experiences through their writing. Additionally, participants continued to use SOVA because they gained a place for self-reflection about their mental health. Third, bloggers found benefits for their mental health in areas of self-esteem, youth competence, and confidence.

### Website Feasibility and Usability

The intervention’s favorable feasibility and usability ratings, combined with the positive feedback in the 3-month surveys, suggest that peer blogging for SOVA is a feasible intervention for AYA who self-reported symptoms of mental illnesses such as anxiety and depression, even without any prior experience in blogging in general or about mental health topics. The site also proved to be usable and acceptable, particularly because of its simplicity and organization and, importantly, its anonymity.

The most common reason participants gave for barriers from blogging was that they were too busy and did not have enough time to go on SOVA, though there were comments that participants wanted to and did use the website when they had free time. Although busyness was a barrier from participants blogging on the website, SOVA can be an outlet for adolescents to manage their stress in their busy schedules, find coping mechanisms, and read articles by peers who may be going through similar stressors. However, because participants are not obligated to blog on the website, they may lose priority in their otherwise busy schedules. Older adolescents and AYA who experience emotional and mental struggles have also been shown to have negative adherence to treatment [50], which may apply to mental health interventions such as SOVA. Our intervention used compensation to show appreciation for content creation, and we found that submissions increased closer to the monthly deadline.

### Reasons for SOVA Blogging

In text-based comments, participants indicated that enjoyment in writing motivated them to continue blogging at the site. In addition, most felt comfortable contributing to the SOVA website because of the anonymity of the website and its moderated nature. Although adolescents are largely on social media, the lack of anonymity on most platforms may prevent them from wanting to open up about their mental health in fear of others judging them because of stigma. Social media platforms that allow anonymity, but do not have moderation, have their own risks, such as cyberbullying [51] and spreading misinformation [52].

SOVA offers a middle ground between sharing information openly and staying anonymous. It does not allow users to have their usernames, blog posts, or comments to include personal information, thereby preventing exposing users to harm by having their content traced back to them. For safety purposes, users’ contact information is collected but stored on a secure university server that is separate from the website. The intervention with the SOVA Peer Ambassador Advisor and moderators still allows users to be open and vocal enough to share openly in a welcoming, safe community.

The results of the intervention also suggest that SOVA serves more than a space for adolescents to discuss their mental health safely and anonymously. On the basis of their reasons for joining and continuing, there is a desire for advocacy and to be a source of peer support for others who may be struggling with their mental health. Participants genuinely wanted to help others because of their own experiences in dealing with problems with their mental health. This corresponds with findings about how younger generations today have a desire to help others and advocate for social justice issues and believe that social media is an effective outlet [53,54].

Given participants’ reasons for blogging, SOVA seems to serve as a platform of social support as well as an outlet of therapeutic writing as a way to manage AYA stressors and symptoms. Interventions such as SOVA find a balance in anonymity and moderation that give users a space to open up without the fear of pushback, cyberbullying, being *exposed* to others they know, and misinformation.

### Effects on Participant Mental Health

Participants had increased self-esteem, competence, and confidence. These findings are similar to other studies that have also found that users participating in adolescent forums to discuss mental health saw an increase in their confidence [55]. There are several reasons that may specifically explain the increase in these three domains in the SOVA intervention.

As previously discussed, participants joined and continued the study because of a desire to help others. Previous research has found similar results in the relationship between helping others and an increase in adolescents’ self-esteem [56]. By helping others, adolescents have a more positive view of themselves, and this positive self-image can lead to increased confidence [56]. In our study, participants were motivated to blog to help their peers, felt that they did so in their responses in the 3-month survey, and thus had an increased positive view of themselves. This may additionally explain the nonsignificant results of the scores measured by the Positive Youth Development Scale. Participants already showed high scores and may have been the reason why they chose to participate in the study. There was little room for these participants to improve.

Most posts written by participants were of a psychoeducational nature. The ability to use their lived experience in the context of psychoeducation to support others—as evidenced to users by affirming comments received to posts they have written—may have changed prior beliefs that their mental illness is a character flaw into the belief that their experiences have provided them with competence in mental wellness.

### Limitations

This study had several limitations. The major limitation is the small sample size and the lack of a control group. Although there were participants who joined and did not blog, nor did they use the website, the low number was not enough for us to make even comparisons between bloggers and nonbloggers at 3 months. There may be limited clinical significance for small to medium effect sizes, which is limited by the small sample size. Another limitation is that multiple comparisons were made using a small sample size. Due to the pilot nature of this exploratory study, we did not want to use methods that were too stringent to erase potentially significant results [57]. Instead, we interpret our findings as a signal to inform measures for future fully powered trials. A larger, more definitive study with a control arm is needed to further examine these findings.

In addition, selection and response bias likely accounted for the low response rate of 72% (34/47) of participants who blogged and completed the 3-month survey. We did not identify any significant differences between those who took the 3-month survey and those who did not. However, because the study is solely web-based with the goal of increasing blogger comfort by sharing personal stories on the internet, this may limit retention as the research team never comes face-to-face with participants. The intervention also had considerable flexibility. For example, the study did not require participants to blog every month. This flexibility may make adherence to the intervention more difficult, albeit more feasible for bloggers who cited a lack of time as a reason they may not blog. As a pilot feasibility study, this study provides groundwork for future studies that include more active recruitment and retention efforts while also including a control group that may reveal increasing severity of symptoms of depression or anxiety in the group not participating in blogging, as symptom severity was maintained at low levels throughout the study, and we cannot ascertain whether this is comparable with a comparison group. The participants were followed for only 3 months. This may be too little time to observe if there are any long-term changes in participants’ mental health. Including additional surveys every 3 months for the first year of participation may be a stronger measure.

Recruitment methods and geographical location may have accounted for the lack of diversity in the study. Adolescents using the Pitt+Me database primarily consist of students attending a predominantly White institution; most of the participants being recruited from the database reflect these demographics. Future recruitment and similar interventions may need to consider more direct community outreach and utilizing outlets such as other social media platforms to find a more diverse sample, as well as a specific implementation intervention to enhance recruitment.

Due to its exploratory nature, this study did not have a complex study design, but the signal in the findings suggests that larger controlled studies with multiple time points and a more diverse sample are warranted.

### Future Directions

Participants used SOVA to help others by sharing their experiences, while also having an outlet for themselves, and a place to write. The aim of the initial work was to understand the feasibility of involving participants in making a substantial contribution to the intervention. Future directions include recruiting a larger, more heterogeneous sample, especially as future studies to examine potential benefits require a larger sample. In particular, future implementation should work to understand how to bring blogging and its potential benefits to those who may not be as comfortable or as enthusiastic about writing as our own sample.

The beneficial effects of this exploratory study included an increase in confidence, self-esteem, and competence. Given these results, blogging of the SOVA websites may be a useful supportive intervention for AYA who have prior experience with mental health treatment and are seeking to maintain the skills and knowledge they have gained while providing peer-based psychoeducation to younger AYA who may have not yet sought help for depression or anxiety. Peer blogging ambassadors can act in a peer support role and provide informal advice through their experiences in their blog posts and responses in comments to others’ blog posts. As SOVA attracted those who enjoyed writing, similar interventions can be used as a volunteering or job opportunity for aspiring young journalists to gain writing experience as well as competence in emotional wellness. Other future developments of SOVA can include a discussion board and more organizational tools to make the variety of topics on the website more accessible. In addition, future interventions can explore ways to increase website activity from participants in a manageable way that does not increase stress on busy schedules.

### Conclusions

SOVA is a web-based intervention designed to create a space for AYA to access mental health information, share their own experiences with mental illness, and interact with others who share their own advice and experiences. The intervention serves as a middle ground between sharing anonymously and sharing openly in a welcoming in-person community. In this process of sharing, AYA seem to show a signal of experiencing benefits to increase their resilience and take something that was a perceived handicap and turn that into a strength. The need for virtual mental health interventions has increased because of COVID-19, particularly in AYA. SOVA has shown that carving out a space on the web for AYA to discuss their mental health journeys and needs not only gives them a community with others they can relate to but can improve their morale and confidence by helping others and themselves.

## Abbreviations

AYA: adolescents and young adults
MHSES: Mental Health Self-Efficacy Scale
PHQ-9: Patient Health Questionnaire-9
PYD-VSF: Positive Youth Development Very Short Form
RSES: Rosenberg Self-Esteem Scale
SOVA: Supporting Our Valued Adolescents
